# West Nile Virus Viremia in Eastern Chipmunks (*Tamias striatus*) Sufficient for Infecting Different Mosquitoes

**DOI:** 10.3201/eid1306.061008

**Published:** 2007-06

**Authors:** Kenneth B. Platt, Bradley J. Tucker, Patrick G. Halbur, Sonthaya Tiawsirisup, Bradley J. Blitvich, Flor G. Fabiosa, Lyric C. Bartholomay, Wayne A. Rowley

**Affiliations:** * Iowa State University, Ames, Iowa, USA; † Chulalongkorn University, Bangkok, Thailand

**Keywords:** West Nile virus, chipmunks, Aedes triseriatus, Aedes vexans, Culex pipiens, research

## Abstract

In eastern chipmunks (*Tamias striatus*) inoculated intramuscularly with 10^1.5^ to 10^5.7^ PFU of West Nile virus (WNV), serum titers developed sufficient to infect *Aedes triseriatus* (Say), *Ae. vexans* (Meigen), and *Culex pipiens* (L.). Mean titers (95% confidence interval) of 8 chipmunks were 10^3.9(3.3–4.5)^, 10^6.7(6.4–7.0)^, and 10^5.8(4.1–7.5)^ PFU/mL on days 1–3 postinoculation (p.i.) and 10^5.8^ PFU/mL in 1 chipmunk on day 4 p.i. Mean estimated days that WNV titers were >10^4.8^ and >10^5.6^ were 1.7 (1.1–2.3) and 1.4 (1.0–1.6). The longest period of viremia >10^4.8^ PFU/mL was 3–4 days. WNV antigen was detected in the small intestine of 2 chipmunks and the kidneys of 4 chipmunks by immunohistochemistry. WNV also was detected in urine, saliva, and feces of some chipmunks. These data suggest chipmunks might play a role in enzootic WNV cycles and be an amplifying host for mosquitoes that could infect humans.

## Abstract 2

Chipmunks might play a role in enzootic WNV cycles and be an amplifying host for mosquitoes that infect humans.

The role of mammals in the ecology of West Nile virus (WNV) has not been well defined. In many mammals, levels of viremia sufficient to infect mosquito vectors do not develop (*1*). However, mosquito-infective viremia levels do occur in some mammals such as the golden hamster (*Mesocricetus auratus*), in which WNV serum titers can exceed 10^5.5^ tissue culture infective dose (TCID)_50_/mL (*2*), and the fox squirrel (*Sciurus niger*), in which a maximum titer of 10^5.0^ PFU/mL was reported (*3*). Mosquito-infective WNV serum titers with a mean duration of 2.2 ± 0.6 days also have been demonstrated in the eastern cottontail rabbit (*Sylvilagus floridanus*) (*4*). Minimum estimated infection rates of 12% and 21% occurred in *Culex pipiens* (L.) and *Cx. salinarius* (Coq.) after they fed on cottontail rabbits with WNV serum titers of 10^4.3^–10^5.0^ TCID_50_/mL. Infection rates increased to 21% and 25% at WNV titers of 10^5.0^–10^6.0^ TCID_50_/mL. The magnitude and duration of WNV viremia levels in cottontail rabbits and fox squirrels, which are peridomestic, raise the question of whether other mammals can serve as WNV-amplifying hosts for mosquitoes in enzootic WNV cycles, with potential for transmission to humans.

This study describes the effect of needle inoculation–induced WNV infection in the eastern chipmunk (*Tamias striatus*). We also demonstrate the potential of chipmunks to serve as a WNV-amplifying host for *Cx. pipiens, Aedes triseriatus* (Say), and *Ae. vexans* (Meigen). These species were selected for the study because they share habitats in common with chipmunks in rural and suburban areas. *Cx. pipiens* is a major WNV enzootic and bridge vector in North America (*5*,*6*). It is ornithophilic but will feed on mammals (*7*–*9*), especially as it shifts its feeding preference during late summer and early fall (*10*). *Ae. triseriatus* and *Ae. vexans* are competent laboratory vectors of WNV (*6*), and this virus has been isolated from field specimens (*11*,*12*). Both species feed primarily on mammals (*6*) but also will feed on avian species (*9*,*13*,*14*).

## Methods

### Cells, Media, Diluents, and Solutions

Vero 76 cells were used for virus propagation, plaque assays, and virus isolation. Growth medium (GM) was Dulbecco modified Eagle medium (GIBCO, Invitrogen Corp., Carlsbad, CA, USA) that contained 10% heat-inactivated fetal bovine serum (FBS) and 2.0 mmol/L L-glutamine. Maintenance medium (MM) was GM with 1% FBS. Overlay medium (OM) for virus assay was 1 part MM and 1 part 2% agarose (Difco, Becton, Dickinson and Co., Sparks, MD, USA), prepared in Hanks’ balanced salt solution (GIBCO, Invitrogen Corp.). Virus diluent was 1 part MM and 1 part CO_2_-independent medium (GIBCO, Invitrogen Corp.) containing 1% FBS. Mosquito grinding diluent (GD) was GM supplemented with 20% FBS. Twenty milligrams of gentamicin sulfate (GentaMax100, Phoenix Pharmaceuticals, Inc., Belmont, CA, USA) was added per 100 mL of all media and diluents. Feeding solution for capillary tubes used for collecting saliva in artificial transmission assays consisted of 5% (w/v) sucrose and 0.5% FBS in 0.15 mmol/L phosphate-buffered saline, pH 7.0.

### Virus and Virus Assay

WNV strain IA02-crow was used in all experiments. The virus was isolated from a crow in Iowa in 2002 and passed 4× in Vero 76 cell cultures (*15*). Virus was assayed by the plaque method. Serial 10-fold dilutions of serum were made in cold (≈4°C) virus diluent. Individual 6-well cell culture plates containing confluent Vero 76 cell monolayers were inoculated in duplicate with 0.75 mL of each virus dilution and incubated for 75 min at 37°C in a 5% CO_2_ atmosphere. The inoculums were replaced with 3 mL of OM, and plates were maintained at 37°C. Three days later, an additional 3 mL of OM containing 2% stock neutral red solution (Sigma Aldrich, Saint Louis, MO, USA) was added to the original overlays. Plates were maintained at 37°C overnight; plaques were then counted and titers were expressed as PFU/mL.

### Experimental Animals

Mature eastern chipmunks were captured with live traps (H.B. Sherman Traps, Tallahassee, FL, USA) in Story County, Iowa, in the fall of 2005 under a state of Iowa permit. Chipmunks were verified to be free of antibodies to WNV by an epitope-blocking ELISA (*16*). Chipmunks were housed in individual cages in the biosafety level 3 animal facility at Iowa State University (ISU) and treated and handled in accordance with guidelines established by the ISU Institutional Animal Care and Use Committee. Chipmunks were anesthetized with ketamine/aceopromazine at 10:1.0 mg/kg for mosquito feeding and blood collection. Chipmunks were bled once from the retroorbital plexus of each eye and once from the heart. Blood was not collected again from the retroorbital plexus of either eye for at least 7 days, or from the heart unless the bleeding was terminal. Two-day-old WNV antibody–free broiler chickens (Hoover’s Hatchery, Inc., Rudd, IA, USA) were used to detect virus transmission by mosquitoes that previously fed on infected chipmunks.

### Mosquitoes

*Ae. triseriatus and Cx. pipiens* were collected in Iowa and colonized in 2002. *Ae. vexans* were first-generation mosquitoes collected in central Iowa during the summer of 2004. Mosquitoes were maintained on 10% sucrose (w/v) in controlled conditions (27°C ± 1°C and 80% ± 5% relative humidity) with a 16:8-hour photoperiod. Mosquitoes were deprived of sucrose for 48 hours and water for 24 hours before feeding on chipmunks and chickens or before artificial transmission experiments.

### Virus Isolation from Mosquitoes

Mosquitoes were killed by freezing. Whole insects, bodies without legs and wings, and legs alone were triturated individually in 1.5-mL microcentrifuge tubes containing 300 μL of cold (≈4°C) GD. For artificial transmission assays, the contents of individual capillary tubes containing saliva of mosquitoes were deposited into 300 μL of GD and stored at –70°C until assayed. Samples were thawed at 37°C and brought to a final volume of 2 mL with cold MM. Approximately 1.6 mL of each sample was passed through a 0.45-μm filter onto a confluent cell monolayer in a 25-cm^2^ cell culture flask. The remaining volume was stored at –70°C. Inoculated flasks were incubated for 1 h at 37°C, and then 5 mL of MM was added. Inoculated cell cultures and controls were observed daily for cytopathic effects (CPE) for up to 7 days postinoculation (p.i.). WNV specificity of CPE was confirmed by the VecTest WNV/SLE antigen assay (Microgenics Corp., Freemont, CA, USA).

### Epitope-blocking ELISA

Serum samples were tested for antibodies to flaviviruses by a blocking ELISA as previously described (*16*). The ELISAs were performed by using the WNV-specific monoclonal antibody (MAb) 3.1112G (Chemicon International, Temecula, CA, USA) or the flavivirus-specific MAb 6B6C-1, obtained from the Division of Vector-Borne Infectious Diseases, Centers for Disease Control and Prevention, Fort Collins, Colorado, USA. The ability of test sera to block binding of the MAbs to WNV antigen was compared to the blocking ability of control sera from chipmunks without antibodies to flaviviruses. Data were expressed as relative percentages and inhibition values; a threshold >30% was considered to indicate viral antibodies.

### Histopathology and Immunohistochemistry

Tissue samples were fixed in 10% formalin and processed for histologic examination by the Veterinary Diagnostic Laboratory at ISU following standardized protocols. WNV-specific mouse ascites fluid (ATCC Catalog #VR01267CAF, Manassas, VA, USA) was used as the primary antibody at a dilution of 1:2,000 to detect WNV antigen. A 30-minute staining period was used. Processed tissues, including negative controls, were evaluated blindly by a veterinary pathologist for evidence of microscopic lesions and scoring of the amount of WNV antigen in the tissues. The amount of WNV antigen in the tissues was given a score of 0 (no staining), + (mild multifocal staining of individual cells), ++ (moderate multifocal staining of cells), or +++ (large amounts of antigen and large numbers of cells staining).

### Characterization of WNV Infection of Eastern Chipmunks

Initially, 1 chipmunk was inoculated intramuscularly (i.m.) with 10^5.7^ PFU of WNV. Blood was collected 2 days later and found viremic for WNV. Subsequently, 6 chipmunks were divided into 2 equal groups. One group was inoculated i.m. with 10^3.5^ PFU of WNV and the other with 10^1.5^ PFU. An eighth chipmunk was subsequently inoculated with 10^2.6^ PFU. Serum specimens for WNV assay were collected on days 1–4 p.i. and then not again until day 8 p.i. If we encountered difficulty in obtaining blood, we aborted the procedure. Swabs of oral and rectal cavities of chipmunks that were inoculated with WNV were taken on days 1–3 p.i. and assayed for WNV. Swabs of urine also were collected from chipmunks when possible.

Tissues—including the brain, spinal cord, lung, heart, skeletal muscle, liver, kidney, spleen, pancreas, small and large intestine, adrenal glands, and salivary glands—were collected from all chipmunks. These tissues were examined for gross and microscopic lesions and assayed by immunohistochemical tests for WNV antigen. Tissues from 1 chipmunk that had not been inoculated served as negative controls.

### Eastern Chipmunks as Source of WNV for Mosquitoes

Groups of up to 15 *Ae. triseriatus*, *Ae. vexans,* and *Cx. pipiens* in the same container were fed on chipmunks between days 1 and 4 p.i. The blood-fed mosquitoes were separated by species, maintained for 14 to 15 days as described previously, and assayed for WNV infection. *Ae. triseriatus* also were assayed for disseminated infections.

*Ae. triseriatus* transmission of WNV was determined by feeding mosquitoes on 2-day-old chickens or by permitting mosquitoes, from which wings and legs were removed, to feed for 20 minutes from capillary tubes containing feeding solution. Transmission was confirmed by detecting WNV in blood that was collected from chickens ≈48 hours after mosquitoes fed, or in the contents of capillary tubes from which mosquitoes fed.

## Results

### Characterization of WNV Infection of Eastern Chipmunks

**Figure 1:**
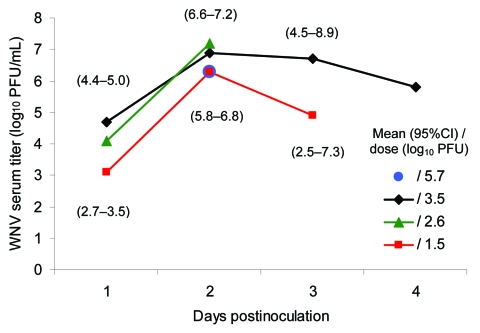
West Nile virus (WNV) viremia profile in 8 eastern chipmunks (*Tamias striatus*) that were inoculated intramuscularly with virus. One chipmunk received 10^5.7^ PFU of WNV and was sampled only on day 2 postinoculation (p.i.). Three chipmunks received 10^3.5^ PFU of WNV, 1 received 10^2.6^ PFU, and 3 received 10^1.5^ PFU. The number of chipmunks that received 10^3.5^ PFU of WNV decreased to 2 on day 3 p.i. and to 1 on day 4 p.i. The number of chipmunks that received 10^1.5^ PFU of WNV was 2 on days 2 and 3 p.i.

WNV viremia developed in all 8 chipmunks (Figure 1) after they were infected with the virus by needle inoculation. Viremia titers peaked on day 2 p.i. and were generally higher in those chipmunks that were inoculated with higher doses of virus. Peak WNV titers of 10^7.2^ and 10^7.8^ PFU/mL of serum occurred in 2 chipmunks that were inoculated with 10^2.6^ or 10^3.5^ PFU of WNV, respectively. The highest titer observed in chipmunks inoculated with 10^1.5^ PFU of WNV was 10^6.5^ PFU/mL. The mean estimated number of days and 95% confidence intervals that WNV serum titers remained >10^4.8^ PFU/mL and >10^5.6^ PFU/mL were 1.7 (1.1–2.3) and 1.4 (1.0–1.6), respectively. The longest period of time that WNV titers were >10^4.8^ PFU/mL was at least 3 days and occurred in a chipmunk that was inoculated with 10^3.5^ PFU of WNV. No WNV was detected in serum specimens from 3 chipmunks that were bled on day 8 p.i.

No WNV was isolated from oral or rectal cavities of infected chipmunks on day 1 p.i., but WNV was isolated on day 2 p.i. from the oral cavities of 4 chipmunks. On day 3 p.i., WNV was isolated from the oral cavities of 4, the rectal cavities of 3, and the urine of 2 chipmunks.

No signs of illness were observed in any of the WNV-infected chipmunks during the first 8 days p.i. Two chipmunks died on days 1 and 2 p.i. during sampling, and a third chipmunk was killed on day 4 p.i. to obtain blood for WNV assay. The first potential signs of WNV infection were observed in the remaining 5 chipmunks between days 9 and 11 p.i. In 3 chipmunks, neurologic symptoms developed, characterized by head tilt and incoordination; the chipmunks were humanely killed. A fourth chipmunk became lethargic and was reluctant to move; it also was humanely killed. A fifth chipmunk had no signs of illness but died unexpectedly on day 27 p.i. WNV-specific antibody was detected in 3 chipmunks that were bled on day 8, 11, or 14 p.i.

**Figure 2:**
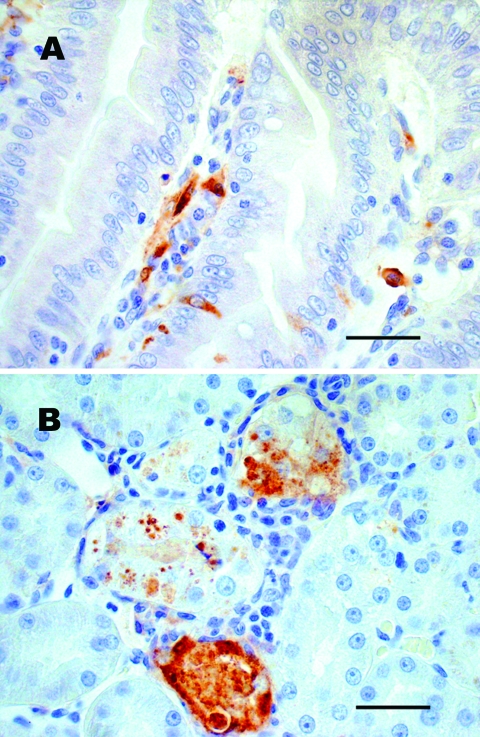
Sections of chipmunk tissues 9 days after intramuscular inoculation with West Nile virus (WNV). A) Lesions were absent, but WNV antigen (brown staining) was demonstrated in scattered epithelial cells and in macrophagelike cells in the lamina propria of the small intestine. B) WNV antigen (brown staining) was demonstrated in necrotic renal tubular epithelial cells. Tissues were stained with hematoxylin, and WNV-specific mouse ascites fluid (ATCC Catalog #VR01267CAF) was used as the primary antibody for immunohistochemical staining. Scale bars = 50 μm.

No gross lesions were observed in any chipmunk; however, microscopic lesions and/or WNV antigen were observed in tissues of 4 of 8 infected chipmunks (Table 1). Mild to moderate multifocal lymphoplasmacytic meningoencephalitis with gliosis and neuronal necrosis was observed in 2 chipmunks on days 9 and 11 p.i. Mild lymphohistiocytic perivascular cuffing of vessels with moderate to severe multifocal hemorrhage throughout the brain was present in 1 of these 2 chipmunks. WNV antigen also was detected in neurons of both chipmunks. Mild lymphoplasmacytic and histiocytic hepatitis were observed in 3 chipmunks. Viral antigen was also detected in Kupffer cells and macrophages of 2 of these 3 chipmunks. Mild multifocal lymphoplasmacytic interstitial nephritis with mild focal renal tubular necrosis was observed in 2 chipmunks on days 9 and 11 p.i. WNV antigen was present in renal tubular epithelial cells and in the renal arterial walls (Figure 2) of these 2 chipmunks and in 2 others in which no kidney lesions were found. WNV antigen in the absence of lesions was demonstrated in the muscularis mucosa, scattered mucosal epithelial cells, and mononuclear cells in the lamina propria of the small intestine of 2 chipmunks.

**Table 1:** Histologic lesions observed in eastern chipmunks (*Tamias striatus*) after intramuscular inoculation with West Nile virus (WNV)

Chipmunk no.	Experiment no.	WNV dose*	Day of death†	Microscopic lesions‡/WNV antigen§¶				
				Brain	Liver	Spleen	Kidney	Small intestine
1	1	5.7	11 (E)	−/−	−/−	−/−	−/−	−/−
2	2	3.5	2 (S)	−/−	−/−	−/−	−/−	−/−
3	2	3.5	11 (E)	++/+++	+/−	−/−	+/+	−/−
4	2	3.5	4 (S)	−/−	+/++	–/+	–/+	–/+
5	2	1.5	27 (U)	−/−	−/−	−/−	−/−	−/−
6	2	1.5	1 (S)	−/−	−/−	−/−	−/−	−/−
7	2	1.5	14 (E)	−/−	−/−	−/−	–/+	−/−
8	3	2.6	9 (E)	++/++	+/++	−/−	+/++	–/++

*Log_10_ PFU of WNV. †Day of death, day postinoculation that death occurred by euthanasia (E) if a chipmunk had symptoms that prevented it from ambulating to or consuming water or food; S, death associated with sampling; U, cause of death not determined. ‡By histologic examination, lesions were not present (–), mild (+), moderate (++), or severe (+++). §By histologic examination, WNV antigen was absent (–), mild (+), moderate (++), or extensive (+++). ¶Organs with no detectable lesions or WNV antigen included heart, lung, adrenal and salivary glands, large intestine, pancreas, and urinary bladder.

### Eastern Chipmunks as a Source of WNV for Mosquitoes

The feeding success rates of *Ae. triseriatus*, *Ae. vexan,* and *Cx. pipiens* on viremic chipmunks were 68%± 5%, 27% ± 6%, and 8% ± 4%, respectively. The WNV infection rates of the 3 mosquito species that fed on these chipmunks are summarized in Table 2. Disseminated infections developed in 5 of the 6 WNV-infected *Ae. triseriatus* that fed on chipmunks with WNV serum titers >10^5.6^ PFU/mL. Two (67%) of 3 *Ae. triseriatus* with disseminated infections transmitted WNV in an artificial transmission assay. One *Ae. triseriatus* with a disseminated infection that fed on a 2-day-old chicken did not transmit WNV.

**Table 2:** Infection rates of *Aedes triseriatus* (Say), *Ae. vexans* (Meigen), and *Culex pipiens* (L.) after feeding on viremic eastern chipmunks (*Tamias striatus*) infected with West Nile virus (WNV)

WNV serum titer*	*Ae. triseriatus*		*Ae. vexans*		*Cx. pipiens*	
	No. blood meals†	% Positive (no. blood-fed)	No. blood meals	% Positive (no. blood-fed)	No. blood meals	% Positive (no. blood-fed)
3.1–4.4	3	0 (27)	2	0 (10)	2	0 (6)
4.8	2	0 (19)	1	0 (1)	1	0 (1)
5.6–5.8	2	23 (13)	2	0 (4)	1	0 (1)
6.0–6.9	5	0 (14)	3	15 (13)	2	50 (2)
7.2–7.8	2	25 (12)	2	40 (5)	ND‡	

*Log_10,_ PFU of WNV. †No. chipmunks providing blood meals. ‡ND, not done.

## Discussion

In chipmunks infected with WNV, viremia titers developed that were sufficient to infect mosquitoes. This finding is noteworthy because the eastern chipmunk is ubiquitous throughout forested, rural, and suburban areas of southeastern Canada, and in the eastern United States, with the exception of the eastern parts of North and South Carolina, Georgia, and most of Florida. Its western range is described by a boundary extending from northwestern North Dakota to southeastern Louisiana (*17*). Because of its high reproductive potential, the eastern chipmunk frequently becomes a pest around homes, gardens, and public parks. Because of the chipmunk’s close association with humans, and its susceptibility to WNV manifested by viremia levels of relatively long duration and high titers (Figure 1), this species, like the cottontail rabbit (*4*), is a potential source of WNV for zoophilic and opportunistic mosquito vectors that have the potential to transmit WNV to humans.

The potential importance of the eastern chipmunk as an amplifying host was demonstrated by the persistence of WNV serum titers >10^4.8^ and 10^5.6^ PFU/mL for average periods of 1.7 (1.1–2.3) and 1.4 (1.0–1.6) days, respectively. These levels of viremia were sufficient to infect Iowa strains of *Ae. triseriatus*, *Ae. vexans*, and *Cx. pipiens* that fed on viremic chipmunks (Table 2). Other investigators also have shown that these 3 mosquito species can become infected by the levels of WNV that occur in chipmunks. The regression model developed by Erickson et al. (*15*) to characterize WNV susceptibility of an Iowa strain of *Ae. triseriatus* predicts an infection rate of 26% after mosquitoes feed on a host with a WNV serum titer of 10^5.6^ PFU/mL. This infection rate is similar to what we observed in the present study (Table 2). WNV infection and transmission rates of 31% and 12% also have been reported for an eastern strain of *Ae. triseriatus* that fed on chickens with titers of 10^7.1^ PFU/mL blood (*6*), a level of viremia that was exceeded in 2 (25%) of the 8 chipmunks infected with WNV in our study. Similarly, WNV infection and transmission rates of 44% and 11%, and 32% and 23%, respectively, have been reported for eastern and western strains of *Ae. vexans* that fed on chickens (*6*) or hanging drops of defibrinated blood (*18*) with titers of 10^7.1^ PFU/mL. Studies in our laboratory also showed infection and transmission rates of 28% and 9% for *Ae. vexans* that fed on chickens with WNV serum titers of 10^5.0^ to 10^5.4^ PFU/mL, respectively (S. Tiawsirisup, unpub. data). These same studies demonstrated WNV infection and transmission rates of 43% and 16% for *Cx. pipiens* that fed on chickens with titers ranging from 10^5.1^ to 10^5.4^ PFU/mL, respectively. WNV infection and transmission rates of 100% and 71%, respectively, also have been reported for a California strain of *Cx. pipiens* that fed on blood with a titer of 10^7.1^ PFU/mL (*18*). These observations suggest that all 3 of these species could be involved in a mosquito-chipmunk-mosquito cycle. However, field studies of host preference for *Cx. pipiens* collected throughout mosquito seasons would be necessary for determining the potential role of *Cx. pipiens* in a mosquito-chipmunk-mosquito cycle.

Other zoophilic and opportunistic mosquito species, commonly found in habitats used by chipmunks, which could be infected by feeding on viremic chipmunks include *Ae. trivittatus* (Coq.), *Ae. albopictus* (Skuse), and *Cx. salinarius*. Cumulative infection and transmission rates of 70% and 24%, respectively, have been reported for an Iowa strain of *Ae. trivittatus* that fed on chickens with WNV serum titers of 10^5.5^ to 10^7.0^ TCID_50_/mL (*19*). Sardelis et al. (*20*) reported WNV infection and dissemination rates up to 53%, and 49% for 3 North American strains of *Ae. albopictus* that fed on chickens with blood titers of 10^5.7^ PFU/mL. Infection and estimated transmission rates of 95% and 34% were reported for a Texas strain of *Cx. salinarius* that fed on chickens with WNV blood titers of 10^6.6^ PFU/mL (*21*), and a minimum estimated infection rate of 21% occurred in *Cx. salinarius* that fed on cottontail rabbits with WNV serum titers of 10^4.3^ to 10^4.9^ TCID_50_/mL (*4*).

Four (80%) of the 5 WNV-infected chipmunks that were maintained beyond day 4 p.i. died. The other 3 chipmunks were not maintained beyond day 4 p.i. The deaths of 2 chipmunks on days 1 and 2 p.i. could be attributed to sampling stress or anesthesia, as observed during field surveys; however, WNV infection may have contributed to their deaths because both animals exhibited WNV viremia.

Microscopic lesions observed in the brain, kidney, and liver in some chipmunks were similar to lesions described in related species such as the golden hamster and the fox squirrel (*2*,*3*,*22*). The absence of detectable lesions or WNV antigen in any tissues of chipmunks 2 and 6 on days 1 and 2 p.i. and the absence of brain lesions or WNV antigen in chipmunks 1 and 7 on days 11 and 14 p.i. were not unexpected (Table 1). Xiao et al. (*2*) did not observe any marked histopathologic changes in the kidney, liver, lung, heart or pancreas of hamsters during the first 10 days p.i., although “spotty splenic necrosis” was observed in some hamsters. However, Xiao et al. did observe the beginning of lesions in many areas of the brain on day 5 p.i. These lesions became more numerous throughout the brain by day 6 p.i. but by day 10 p.i. were found mostly in the brain stem. Thus, detectable lesions might have been present earlier in the brain of chipmunks 1 and 7 but diminished to undetectable limits by days 11 and 14 p.i.

The presence of WNV in the urine, saliva, and rectum of some chipmunks raises the question of whether chipmunks can be infected by the fecal-oral route. Oral infection by WNV has been documented in the golden hamster (*23*), the American alligator (*Alligator mississippiensis*) (*24*), the domestic cat (*Felis catus*) (*25*), and some raptors (*26*). Nonmosquito transmission from infected to naive chipmunks might be a mechanism that could contribute to the maintenance of WNV between epidemic periods, particularly if WNV establishes a persistent infection in chipmunks. Recently, Tesh et al. (*27*) described persistent WNV infection in golden hamsters, which shed WNV in urine for up to 247 days p.i. in the presence of serum neutralizing antibody. WNV also has been isolated from kidney tissue of rhesus macaques (*Macaca mulatta*) 5.5 months after infection (*28*). WNV RNA also has been isolated from the spleen, lung, and kidney of birds 6 weeks after experimental infection (*29*). These findings indicate that WNV can persist in some mammals and birds. The presence of WNV antigen in kidney tissue of a chipmunk at 14 days p.i. (Figure 2) in the presence of antibody could indicate that persistent WNV infections also occur in chipmunks. Whether persistent infection occurs and how long it remains are subjects of further study.

Additional study to determine the role of the chipmunk in the ecology of WNV is justified for the following reasons: 1) the high levels of viremia in chipmunks after injection of WNV doses that can be delivered by a variety of mosquitoes (*30*,*31*), 2) the WNV vector competence of several zoophilic and opportunistic mosquitoes that share the same habitats with chipmunks, and 3) the possibility of persistent infection. These studies should include characterizing the profile of the WNV viremia after infection by mosquito bite because the onset and level of viremia can be affected by components in mosquito saliva (*32*). Determining the seasonal seroprevalence of antibodies to WNV in chipmunk populations in WNV-endemic areas and mortality rates after natural infection also would provide an indication of the relative importance of the chipmunk in the ecology of WNV.
